# Bürger:innen als Frühwarnsysteme (Public Health Sentinels) in der öffentlichen Gesundheit: Fokusgruppen zur Teilnahme an einer adaptiven und kontextsensitiven Echtzeitkohortenstudie in Krisenzeiten

**DOI:** 10.1007/s00103-025-04108-3

**Published:** 2025-07-28

**Authors:** Stephanie Zintel, Hannah Z. Hennig, Christopher M. Jones, Vera Araújo-Soares, Marike Andreas, Kristina Hoffmann, Birgit Kramer, Björn Mergarten, Sven Schneider, Falko F. Sniehotta, Anna K. Kaiser

**Affiliations:** Zentrum für Präventivmedizin und Digitale Gesundheit (CPD), Medizinische Fakultät Mannheim, Universität Heidelberg, Röntgenstr. 7, 68167 Mannheim, Deutschland

**Keywords:** Studienteilnahme, Kohortenstudie, Verhaltensdeterminanten, Theoretical Domains Framework, Implementierung, Teilnahmebereitschaft, Study participation, Cohort study, Behavioral determinants, Theoretical Domains Framework, Implementation, Willingness to participate

## Abstract

**Hintergrund:**

Die Covid-19-Pandemie hat den Bedarf an hochauflösender Evidenz zu sozialen und kontextuellen Bedingungen sowie den gesundheitlichen Folgen solcher Ausnahmesituationen deutlich gemacht. Mit der PULS(Populationsbasierte Umfrage zur Lebenssituation und Sozialen Gesundheit)-Studie soll deshalb eine agile und responsive Echtzeitkohorte etabliert werden. In einem ersten Schritt sollen die Determinanten der Studienteilnahme analysiert werden.

**Methoden:**

Qualitative Studie im Zeitraum 16.12.2024–14.02.2025 mit 7 leitfadengestützten Fokusgruppen mit jeweils 4–7 Teilnehmenden (insg. 22 männlich, 17 weiblich, 2 nonbinär). Aussagen wurden entlang der 14 Domänen des „Theoretical Domains Framework“ deduktiv codiert.

**Ergebnisse:**

Die Teilnehmenden betonten vor allem die Wichtigkeit verschiedener Aspekte, die den Domänen *Verstärkung, Erwartungen über Auswirkungen* und *Umweltbedingungen/Ressourcen* sowie *Ziele* und *Überzeugungen über Fähigkeiten *zuzuordnen sind. Dabei wurden die einzelnen Aspekte nicht isoliert betrachtet, sondern gegeneinander abgewogen. So waren Teilnehmende beispielsweise eher bereit, zeitliche Ressourcen zu investieren, wenn sie ihren Beitrag zum Gemeinwohl als besonders relevant empfanden – etwa weil ihre persönlichen Ziele mit den politischen und gesellschaftlichen Implikationen der Studie übereinstimmten.

**Diskussion:**

Die Teilnahme an einer agilen und langfristig angelegten Kohortenstudie wird unter bestimmten Bedingungen als akzeptabel und umsetzbar eingeschätzt. Insbesondere der wahrgenommene Einfluss auf Gesellschaft und Politik sowie die Aussicht auf eine wirksame Translation wissenschaftlicher Erkenntnisse in politische Maßnahmen können Schlüsselanreize für Bürger:innen sein, die den persönlichen Ressourceneinsatz aufwiegen. Die Ergebnisse haben nicht nur Bedeutung für die Implementierung der PULS-Studie, sondern auch für anschließende Translationsvorhaben.

**Zusatzmaterial online:**

Zusätzliche Informationen sind in der Online-Version dieses Artikels (10.1007/s00103-025-04108-3) enthalten.

## Abstract (en)

**Background:**

The COVID-19 pandemic has highlighted the need for high-resolution evidence on the social and contextual conditions as well as health outcomes of such emergency events. The PULS (Populationsbasierte Umfrage zur Lebenssituation und Sozialen Gesundheit, i.e., Population-Based Survey on Living Conditions and Social Health) study aims to provide such evidence with an agile and responsive real-time cohort. Here, we examine the determinants of participation in such a study.

**Methods:**

A qualitative study using seven semi-structured focus groups (between 16 December 2024 and 14 February 2025), each with 4 to 7 participants (total of 22 men, 17 women, 2 non-binary individuals; diverse backgrounds). Statements were deductively coded along the 14 domains of the Theoretical Domains Framework.

**Results:**

Participants primarily emphasized the importance of various aspects associated with the domains of *Reinforcement*, *Beliefs about Consequences*, *Environmental Context and Resources*, and *Goals,* and *Beliefs about Capabilities*. However, participants did not only mention distinct aspects but also weighed them against each other. For example, participants were more willing to invest resources such as time if the resulting political and social implications of the study aligned with their personal goals, thus demonstrating a subjective contribution to the common good.

**Discussion:**

Participation in an agile, responsive, and long-term cohort study is viewed acceptable and feasible under certain conditions. The perceived impact on society and politics as well as the translation of research into policy seem to be key incentives for citizens and can outweigh personally invested resources. These results have implications not only for the implementation of the PULS study but also for the related translation pathways.

## Hintergrund

Der Bedarf an einer besseren, evidenzbasierten Vorbereitung des Gesundheitssystems auf plötzlich auftretende Ausnahmesituationen wie die Covid-19-Pandemie, Hitzeperioden oder Naturkatastrophen ist in den vergangenen Jahren deutlich geworden. Derzeit bestehen jedoch Defizite in der strukturierten Erhebung hochauflösender Evidenz zu den gesundheitlichen Folgen solcher Ereignisse.

Agile Echtzeitkohorten bieten die Möglichkeit, zeitnah zu verstehen, wie die Bevölkerung auf externe Einflüsse und gesundheitspolitische Maßnahmen reagiert. Während der Covid-19-Pandemie hat in Deutschland insbesondere die COSMO-Studie mit ihren regelmäßigen Befragungswellen den Wert einer kontinuierlichen, krisenbegleitenden Beobachtung der Bevölkerungsgesundheit unterstrichen. Traditionelle Panel-Designs ermöglichen durch ihre Regelmäßigkeit die Erfassung langfristiger Trends. Allerdings fehlt in der deutschen Forschungslandschaft bislang eine Kohorte, die eine Echtzeiterhebung von gesundheitsspezifischen Daten erlaubt und Veränderungsprozesse sowie die Bedarfe der Bevölkerung in Krisenzeiten hochauflösend und ad hoc erfasst. Erste Konzepte für eine agile Echtzeitkohorte befinden sich bereits in der Entwicklung [1, 2].

Als *Proof of Principle* für eine responsive Echtzeitkohorte plant das Zentrum für Präventivmedizin und Digitale Gesundheit (CPD) an der Universität Heidelberg ein Pilotprojekt (PULS – **P**opulationsbasierte **U**mfrage zur **L**ebenssituation und **S**ozialen Gesundheit), das neben gängigen Gesundheits- und Verhaltensparametern auch kontextuelle und soziale Determinanten der physischen, mentalen und sozialen Gesundheit der Bevölkerung in Krisenzeiten verzögerungsfrei beobachtbar macht.

Ein zentrales Merkmal der Kohorte ist ihre agile, adaptive und responsive Erhebungsstruktur. Teilnehmende stimmen einer App-basierten Datenerhebung zu, die sowohl regelmäßige Erhebungen in festen Abständen als auch situativ intensivierte Befragungen bei vorhersehbaren (z. B. Hitzewellen, Überflutungen, Fluchtbewegungen) und unvorhersehbaren Krisen (z. B. Terroranschläge, Pandemien) umfasst. Die Studie ist so konzipiert, dass sie kurzfristig auf Entwicklungen reagiert und sowohl Inhalte als auch Umfang der Erhebungen flexibel an die jeweilige Situation angepasst werden können. So erfordern etwa Hitzewellen und Pandemien unterschiedliche zeitliche Reaktionsfenster und gesundheitliche Schwerpunkte: Während Hitzewellen oft kurzfristig vorhersehbar sind und primär kardiovaskuläre Belastungen betreffen, entwickeln sich Pandemien langsamer, mit plötzlich steigendem Ausmaß, und bringen andere, krankheitsspezifische Symptomatiken in den Fokus.

Ein weiteres zentrales Element der PULS-Studie ist ein gemeinsames Rollenverständnis (Identität) der Teilnehmenden als *Public Health Sentinels*. Mit diesem Begriff bezeichnen wir Bürger:innen, die durch ihre kontinuierliche Teilnahme aktiv zur öffentlichen Gesundheit beitragen, indem sie gewissermaßen als Frühwarnsystem für gesundheitliche Entwicklungen fungieren. Als „Augen und Ohren“ der öffentlichen Gesundheit helfen sie dabei, relevante Gesundheitsentwicklungen in Echtzeit sichtbar zu machen und so evidenzbasierte Maßnahmen zu ermöglichen.

Allerdings können bestimmte Herausforderungen die Teilnahme erschweren, darunter (1) intensive Erhebungsphasen während ohnehin belastender Ereignisse, (2) der Wechsel zwischen hochfrequenten und weniger intensiven Phasen sowie (3) die langfristige Studiendauer. Um eine nachhaltige Teilnahme sicherzustellen, müssen daher die Determinanten der Beteiligungsbereitschaft frühzeitig gemeinsam mit der Zielgruppe analysiert und in die Studiengestaltung integriert werden.

Obwohl es bisher keine Forschung zur Teilnahmebereitschaft an Echtzeitkohorten gibt, existieren einige Studien zu App-basierten Gesundheitsstudien. Diese identifizieren wichtige Barrieren und Anreize für die Teilnahme, die meist anhand von Umfragen und Vignettenexperimenten gegeneinander abgewogen und mit der hypothetischen Teilnahmebereitschaft korreliert werden.

Zu den Anreizen zählen das Bewusstsein für die wissenschaftliche Relevanz von Daten sowie die Überzeugung, mit der Teilnahme einen Beitrag zum Gemeinwohl zu leisten [3, 4]. Das Vertrauen in den Nutzen und die Integrität universitärer Forschung spielt eine wichtige Rolle, wobei Universitäten als Studienverantwortliche größeres Vertrauen als staatliche Institutionen oder Privatunternehmen genießen [3, 5, 6]. Eine Vergütung steigert generell die Teilnahmebereitschaft an App-basierten Studien [3, 7, 8]. Neben monetärer Vergütung wurden auch andere Anreize wie Feedback untersucht: Während Teilnehmende personalisiertes Feedback als positiv bewerteten, hatte es keinen Einfluss auf die tatsächliche Teilnahme [9]. Hingegen wirkte sich die Möglichkeit, die Datenerhebung aktiv zu steuern, förderlich auf die Teilnahmebereitschaft aus [10]. Datenschutzbedenken können die Teilnahmebereitschaft verringern [3, 7, 11]. Dies zeigten sowohl Vignettenstudien als auch offene Befragungen, in denen sie am häufigsten als Barriere genannt wurden [3, 11]. Auch mangelnde Smartphone-Routine kann eine Barriere darstellen [12]. Generell sank die Bereitschaft zur Teilnahme, wenn eigens für die Studie eine App heruntergeladen werden musste [13].

Ziel dieser Arbeit war es, Barrieren und Anreize für die Teilnahme an einer adaptiven, kontextsensitiven Echtzeitkohortenstudie (PULS-Studie) zu identifizieren. Dabei wurde insbesondere untersucht, wie das spezifische Studiendesign akzeptiert sowie die geplante aktive Rolle der Teilnehmenden als „Augen und Ohren“ der öffentlichen Gesundheit (*Public Health Sentinel*^1^*) *wahrgenommen wird.

## Methoden

Zusammensetzung der Fokusgruppen: Es wurden 6–9 Fokusgruppen anvisiert. Basierend auf Analysen von Guest et al. [14] erwarteten wir eine Datensaturierung bei etwa 6 Fokusgruppen. Am Ende wurden 7 Fokusgruppen durchgeführt, 5 in Präsenz am CPD der Universität Heidelberg und 2 online über MS Teams. Jede Sitzung war für 1,5 h geplant und wurde jeweils von 2 Moderator:innen (HZH (w), KH (w), AKK (w), BK (w), BM (m), SZ (w)) geleitet. Nur in der jeweils ersten Präsenz- und online Fokusgruppe gab es noch eine Beobachterin. Genaue Angaben zur Dauer der Fokusgruppen finden sich in Tabelle S1 im Onlinematerial. Die Gruppen umfassten jeweils 4–8 Teilnehmende, insgesamt nahmen 42 Personen (22 Männer, 18 Frauen, 2 nonbinäre Personen) von insgesamt 85 Interessierten teil. Rückmeldung zur Nichtteilnahme bezogen sich überwiegend auf terminliche Einschränkungen oder eine nicht realisierbare Anreise. Letzteres war auch der Grund dafür, 2 Fokusgruppen online anzubieten.

Die Teilnehmenden waren zwischen 19 und 82 Jahre alt (*M* = 42,4; *SD* = 19,37). 30 hatten Abitur, eine fachgebundene Hochschulreife oder Fachhochschulreife, 3 einen Haupt‑/Volksschulabschluss und 7 einen Realschulabschluss/Mittlere Reife. Die berufliche Zusammensetzung war heterogen, eine Auflistung findet sich in Tabelle S1 im Onlinematerial. Eine gezielt rekrutierte *Community-Leader-Gruppe* – also Personen mit besonderer gesellschaftlicher Verantwortung und Multiplikator:innenrolle in ihrem lokalen Umfeld – bestand aus Vertreter:innen sozialer Berufe und des öffentlichen Dienstes (Bürgermeister:innen, Quartiersmanager:innen, Schulleitungen von Förderschulen, Leitungen von Seniorenbegegnungsstätten, Freiwillige Feuerwehr).

Die Rekrutierung erfolgte über persönliche Ansprache im öffentlichen Raum Mannheims (Supermarkt, Innenstadt, Marktplatz). Einzelne Teilnehmende wurden über persönliche Kontakte – im direkten Gespräch oder über Smartphone-Kommunikation – auf die Studie aufmerksam gemacht. Eine öffentliche Ausschreibung wurde bewusst vermieden, um eine Verzerrung durch besonders forschungsaffine Teilnehmende zu minimieren.

Interessierte füllten einen Screening-Fragebogen zu Bildungs- und Berufsstatus aus und wurden anschließend telefonisch oder per E‑Mail eingeladen. Die Fokusgruppen fanden vom 16.12.2024 bis 14.02.2025 statt. Die Teilnahme wurde mit 50 € vergütet, um sonst selten erreichte Gruppen zur Teilnahme zu motivieren. Alle Fokusgruppen wurden über MS Teams als Video aufgenommen. Eine zusätzliche Audioaufnahme erfolgte ab der zweiten Fokusgruppe über ein Diktiergerät.

Da die Auswertung ab der vierten Fokusgruppe zeigte, dass im Hinblick auf die Beantwortung der Fragestellung eine inhaltliche Sättigung erreicht war, wurden keine weiteren Fokusgruppen durchgeführt.

### Interviewleitfaden

Jede Fokusgruppe begann in einer lockeren Atmosphäre, in der Studienziele, Einwilligung und rechtliche Fragen geklärt wurden. Für das Gespräch wurde eine Ansprache mit Vornamen und „Sie“ vereinbart, auch gegenüber den Moderator:innen. Es wurde eine Eisbrecherfrage gestellt, die nach den ersten beiden Fokusgruppen geändert wurde. An der ersten Eisbrecherfrage (Lieblingsessen) beteiligten sich die Moderator:innen noch selbst, nicht aber an der zweiten (Erfahrungen während der Covid-19-Pandemie). Um eine einheitliche Gesprächsgrundlage zu schaffen, wurden zu Beginn 2 Videos präsentiert. Ein Video stellte die Ziele der Studie und die Idee des *Public Health Sentinel* dar. Ein weiteres Video veranschaulichte die Eigenschaften der Studie (z. B. Befragung mittels App; 4 reguläre Befragungen von 5–15 min pro Jahr; Fragen zu gesundheitlichen Themen; Module zu sozialen Netzwerken; spontane und intensivere – bis zu tägliche – Befragungen bei Ereignissen wie Hitze- oder Krankheitswellen).

Im Anschluss wurden die folgenden Unterthemen adressiert:

- Einstellungen zur Teilnahme an App-basierten Gesundheitsstudien,
- Akzeptanz einer Identität als *Public Health Sentinel* in gesundheitlichen Krisen,
- Beweggründe, Barrieren und Anreize für eine Teilnahme,
- Einstellungen des Freundes- und Bekanntenkreises.

Der letzte Punkt geht dabei zurück auf das Prinzip des *Human Social Sensing*, das die ausgeprägten Fähigkeiten von Menschen, ihr soziales Umfeld einzuschätzen, nutzt, um soziale Trends zu beschreiben und zu erkennen [15, 16].

Das Studiendesign sah eine kontinuierliche Analyse der Ergebnisse sowie eine iterative Optimierung des Interviewleitfadens und der Videos vor. Nach den ersten beiden Fokusgruppen wurden Anpassungen vorgenommen. Wie oben beschrieben, wurde die ursprüngliche Eisbrecherfrage abgeändert, da sie bei den Teilnehmenden aus unserer Sicht zu viele allgemeingültige Aussagen und zu wenig Bezüge zur persönlichen Erfahrungswelt aktivierte. Die Frage nach den Erfahrungen während der Covid-19-Pandemie verbesserte die Gesprächsdynamik. Zudem wurden die Videos überarbeitet, um die Studienziele klarer zu vermitteln. Danach war keine weitere Überarbeitung notwendig. Der vollständige Interviewleitfaden befindet sich im Onlinematerial. Die Videos können in einem öffentlichen Verzeichnis^2^ des Open Science Framework (OSF) eingesehen werden.

### Forscherteam

Die Interviews wurden von 6 verschiedenen Personen in Zweierteams durchgeführt: HZH (Psychologin), KH (Biologin), AKK (Soziologin), BK (Gerontologin), BM (Biologe) und SZ (Psychologin). Einzelne Interviewer:innen standen in persönlichem Kontakt zu einigen Teilnehmenden, die Leitung der jeweiligen Fokusgruppen übernahmen jedoch stets Interviewer:innen, die den Teilnehmenden nicht persönlich bekannt waren.

AKK und SZ leiten das Forschungsprojekt zur PULS-Studie. Ihre Motivation speiste sich insbesondere aus der Herausforderung, eine vielfältige und divers zusammengesetzte Personengruppe für die Studie zu gewinnen. Die übrigen Moderator:innen waren vor allem durch das Anliegen motiviert, an einem Forschungsprojekt mitzuwirken, das Bürger:innen nicht nur als Forschungsobjekte betrachtet, sondern sie aktiv einbezieht.

### Auswertungsstrategie

Die Aufnahmen der Fokusgruppen wurden transkribiert und mittels MAXQDA (VERBI Software, 2021) kodiert. Aus Zeitgründen konnten weder die Transkripte noch die Ergebnisse an die Teilnehmenden für Feedback weitergeleitet werden.

Die Kodierung erfolgte deduktiv nach den 14 Domänen des Theoretical Domains Framework (TDF), einem integrativen Framework zur systematischen Analyse von Verhaltensänderungen [17].

Verwendet wurde die von Cane et al. [17] validierte zweite TDF-Version, die folgende Domänen umfasst: *Wissen; Fähigkeiten; soziale/berufliche Rolle und Identität; Überzeugungen über Fähigkeiten; Optimismus; Erwartungen über Auswirkungen; Verstärkung; Intentionen; Ziele; Gedächtnis, Aufmerksamkeit und Entscheidungsprozesse; Umweltbedingungen und Ressourcen; soziale Einflüsse; Emotionen; Verhaltensregulationen.* Das Kodier-Manual findet sich im Onlinematerial in Tabelle S2. Für jede Fokusgruppe wurden die Aussagen von 2 Forschenden unabhängig kodiert. 3 Personen waren aktiv an der Kodierung beteiligt (HZH, SZ, CJ), regelmäßige Rücksprachen erfolgten auch mit AKK. Die Intercoder-Reliabilität (ICR) wurde stichprobenartig überprüft, aber nicht systematisch bestimmt. Unklare Kodierungen wurden zunächst im Austausch der Kodierenden diskutiert (HZH, SZ, CJ). Falls kein Konsens gefunden wurde, wurde AKK als leitende und von der Kodierung unabhängige Person hinzugezogen [18]. Da ein zentrales Erkenntnisinteresse darin bestand, Abwägungsprozesse inhaltlich nachzuvollziehen und die darin einfließenden Aspekte zu erfassen, war es auch beabsichtigt, thematische Überlappungen zwischen Codes zuzulassen. Diese konzeptuellen Überlappungen führten jedoch somit auch zu Unschärfen bei der Kodierung, die eine Bewertung auf Grundlage der ICR wenig sinnvoll erscheinen ließen, diesen konnten wir aber stattdessen diskursiv begegnen.

## Ergebnisse

**Abb. 1:**
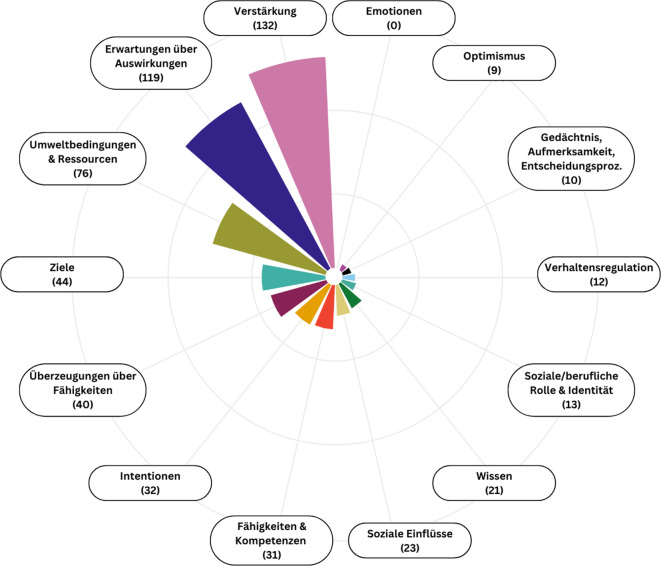
Absolute Häufigkeiten der in den Fokusgruppen genannten Aspekte aus den Domänen des Theoretical Domains Framework (TDF), dargestellt in einem Radardiagramm

Viele Aussagen der Fokusgruppenteilnehmenden ließen sich den Domänen *Verstärkung *(132 Nennungen), *Erwartungen über Auswirkungen *(119 Nennungen) und *Umweltbedingungen und Ressourcen* (76 Nennungen) zuordnen. Auch auf *Ziele *(44 Nennungen), *Überzeugungen über Fähigkeiten *(40 Nennungen), *Intentionen* (32 Nennungen) und *Fähigkeiten *(31 Nennungen) wurde häufig Bezug genommen. Seltener drehten sich Aussagen um *soziale Einflussfaktoren *(23 Nennungen), *Wissen *(21 Nennungen), *soziale/berufliche Rolle und Identität *(13 Nennungen), *Verhaltensregulation *(12 Nennungen)*, Gedächtnis, Aufmerksamkeits- und Entscheidungsprozesse* (10 Nennungen) und *Optimismus *(9 Nennungen; Abb. 1). Im Folgenden werden zentrale inhaltliche Aspekte dieser Themen ausführlicher dargestellt. Dabei liegt ein besonderes Augenmerk auf den komplexen Abwägungsprozessen der Teilnehmenden.

Die Einordnung in die TDF-Domänen zeigte thematische Überschneidungen, weshalb im Folgenden zusammenhängende Themen gemeinsam dargestellt werden. Einzelne Aspekte, wie etwa Bedenken, ob die wichtige Rekrutierung von Personen aus marginalisierten Gruppen, die für eine Repräsentativität der Studienpopulation zentral sind, gelingen könne, oder Zweifel an der eigenen Fähigkeit, Aussagen über andere zu treffen, werden im Folgenden nicht vertiefend dargestellt, da ihre umfassende Analyse den Rahmen der vorliegenden Arbeit überschreiten würde. Eine vollständige Darstellung der Aussagen der Teilnehmenden findet sich im Onlinematerial.

### Ziele und Erwartungen

Die Befragten nannten verschiedene *Ziele*, die sie zu einer Teilnahme an einer App-basierten Gesundheitsstudie motivieren würden. Diese *Ziele* wurden häufig direkt mit *Erwartungen über Auswirkungen* verknüpft. Sie lassen sich danach unterscheiden, ob ein gesellschaftlicher oder ein persönlicher Nutzen angestrebt wird.

#### Gesellschaftlicher Nutzen

Für einige Teilnehmende war insbesondere der Aspekt, etwas zum Gemeinwohl beitragen zu können, sowohl ein essenzielles *Ziel* als auch eine *Erwartung* an die App-basierte Gesundheitsstudie. Eine Person erklärte:

„Der Nachwelt was [mitgeben], dass die halt auch wirklich [damit] was anfangen können. Also ich gebe meinen Nächsten sozusagen was mit, was die noch weiter ausbauen können und vielleicht noch unsere Defizite, was wir jetzt vielleicht machen werden, noch mal einheimsen und es noch besser machen, damit die Zukunft dann noch mehr Erleichterung hat, dass es noch weniger Herzinfarkte oder sonst irgendwas gibt.“

Die Befriedigung durch die Erfüllung dieses *Ziels* und die Wahrnehmung, dass die Teilnahme eine Art Bürgerpflicht darstelle, wurden beispielsweise in folgenden Aussagen deutlich:

„Also die Möglichkeit, eine Rückmeldung zu geben und dass also, wenn ich Vertrauen hätte, dass die Erkenntnisse der Befragung der Allgemeinheit dienen, dann würde ich sagen, ist doch super im Sinne der Basisdemokratie. … Das wäre meine Befriedigung. Dann kann ich was Gutes tun.“

Und:

„Ja, ich finde es gut, dass ich als Bürgerin zu etwas dazu beitrage. Also auch irgendwo eine Pflicht, meiner Meinung nach.“

Auch wurde die *Erwartung* genannt, dass durch die Teilnahme die eigene Stimme gehört und ein Einfluss auf die Studienergebnisse genommen werden könne. Eine Person beschrieb es folgendermaßen:

„Da wird meine Stimme und Erfahrung mitgezählt und mitgedacht und gesehen. Weil, ich würde sagen, das ist eher seltener. Also das ist eher selten, dass es mir so geht und ich würde mir so eine Erfahrung daraus erhoffen.“

In Zusammenhang damit wurde das konkrete *Ziel* geäußert, durch Partizipation Einfluss auf die Politik nehmen zu können. Befragte erhofften sich durch eine Teilnahme eine Verbesserung politischer Maßnahmen.

Die *Erwartungen* eines gesellschaftlichen Nutzens wurden jedoch auch kontrovers diskutiert. Teilnehmende stellten den tatsächlichen Einfluss der Studie infrage und formulierten konkret, dass ihre Teilnahme stark an das *Ziel* eines *sichtbaren* Einflusses gekoppelt sei. So sagte ein:e Teilnehmer:in:

„Also wenn man die ganzen Daten dann hat, was wird umgesetzt? Geht es dann an die Politik, wenn man denen irgendwie Handlungsrichtlinien oder irgendwie [gibt] oder geht es an die Krankenkassen, dass die Präventivmaßnahmen umsetzen? Da würde ich mich fragen, was das bringt überhaupt. Also gar nicht mal die Auswertung an sich, sondern … am Schluss, wo das hingeht und was da rauskommt.“

Hier wurde Frustration gegenüber Politik und Wissenschaft deutlich. Es gab aber auch Teilnehmende, die optimistisch waren, dass die Ergebnisse einer solchen Studie einen positiven Einfluss haben könnten. Diskutiert wurden beispielsweise die Verbesserung des Gesundheitssystems, Meldungen über Krankheiten in abgelegenen Orten, Gesundheit am Arbeitsplatz, Vorbereitung auf Krisensituationen, Weitergabe von Informationen in die Politik und an Krankenkassen.

Eng verbunden mit der *Erwartung* eines tatsächlichen Einflusses der Ergebnisse auf beispielsweise politische Entscheidungsprozesse wurde über die praktische Aussagekraft der Studienergebnisse nachgedacht – insbesondere im Hinblick auf Aspekte des Studiendesigns wie der Frage nach der Repräsentativität, z. B.:

„Also sprich, wie viele Menschen daran mitmachen oder … bereit wären mitzumachen … so dass es wirklich repräsentativ ist im Sinne von … die Möglichkeit haben alle was dazu zu sagen, … dass es nicht wirklich eine schmale Bevölkerungsgruppe ist ... die ihren Senf abgibt, der dann als repräsentativ wahrgenommen wird.“

#### Persönlicher Nutzen

Zusätzlich wurde eine Reihe von *Zielen* mit persönlichem Nutzen genannt. Teilnehmende erwarteten positive persönliche Konsequenzen durch eine Teilnahme an der PULS-Studie, etwa Einblicke in die eigene Gesundheit, Verhaltensverbesserung durch Tracking oder Tipps der App, soziale Vergleichsmöglichkeiten oder ein Gefühl von Unterstützung und Verbundenheit in Krisenzeiten:

„… dass man sieht, … wenn [es] vergleichbare Werte gibt, die schnell in der App angezeigt werden könnten, dass man dann auch sieht mir geht so und anderen geht’s auch so, dass man dann dadurch vielleicht auch den Mehrwert hat, dass man so ein bisschen Gesellschaft mitbekommt und sich wohler fühlt und nicht allein fühlt.“

Einige Teilnehmende teilten jedoch mit, dass sie eben keine persönliche Relevanz und Konsequenz in einer Teilnahme sähen, was sie demotivieren würde.

### Anreize und Motivation zur langfristigen Teilnahme

In der Domäne *Verstärkung *– also Faktoren, die die langfristige Teilnahme fördern – wurde betont, wie wichtig Erinnerungsfunktionen und motivierende Elemente sind. Als Anreize nannten die Teilnehmenden die erwarteten gesellschaftlichen und persönlichen Auswirkungen der Studie. Essenziell sei jedoch, dass diese bereits während der Studiendauer sichtbar werden. Wiederholte Rückmeldungen zur gesellschaftlichen Relevanz und Sinnhaftigkeit der Teilnahme wurden teils als wichtiger empfunden als individuelle Belohnungen. Eine Person beschrieb es wie folgt:

„Wenn ich das Ergebnis meiner Teilnahme im Verlauf der zwei Jahre an anderen Stellen wahrnehme. Und da das Gefühl habe, ich bewirke wirklich was. [Das] wäre mir persönlich jetzt wichtiger noch als … ein Fleißbienchen, was ich einmal im Jahr zugeschickt bekomme. …“

Eine weitere Person beschrieb, wie sie der konkrete Einfluss von Zwischenergebnissen auf die Politik motivieren würde:

„… ich glaube, wichtiger wäre für mich, dass ich das Gefühl habe Ah, das hat wirklich eine Relevanz … Ich sehe das irgendwo umgesetzt. Also meinetwegen … wenn eine Hitzewelle ist oder so, … dass ich das widergespiegelt kriege medial. Dass das irgendwie einen Einfluss in konkretes politisches Handeln oder Handeln von Institutionen dann hat.“

Belohnungen in Form von Geld, Gutscheinen (z. B. für Supermärkte oder Apotheken) oder anderen Geschenken waren nicht nur Gründe für die Anmeldung an einer Studie, sondern auch für die Fortsetzung der Teilnahme. Die Befragten diskutierten dabei auch verschiedene Anreizsysteme als *Verstärker*, z. B. das wiederholte Sammeln von Belohnungen (wie bei einem Bonusprogramm).

### Datenschutzbedenken als Teilnahmebarriere

Im Bereich der *Erwartungen über Auswirkungen* spielte der Datenschutz in allen Gruppen eine Rolle. Er wurde sowohl als persönliche Teilnahmebarriere als auch als Hindernis im Freundes- und Bekanntenkreis wahrgenommen. Eine teilnehmende Person formulierte es folgendermaßen:

„Also mir persönlich würde es besser gefallen, dass es eine Uni ist als die Bundesregierung. Nichts gegen die Bundesregierung. Aber das sind wechselnde … Spieler. … Und mir persönlich wäre das, glaube ich, sympathischer, wenn das an der Uni angesiedelt wäre.“

Allerdings gab es erhebliche Bedenken dahin gehend, ob universitäre Strukturen in der Lage seien, die Daten, z. B. gegen Hacker oder andere externe Zugriffe zu schützen. Einige Teilnehmende äußerten vor allem Bedenken, dass die Daten auf gesamtgesellschaftlicher Ebene missbraucht werden könnten,

„dass jemand halt aktiv nach Anzeichen von … queer oder transgender Menschen sucht oder auch geistigen Störungen und daran halt Hasskriminalität ausübt. Wenn das auch je nachdem wie sich diese App entwickeln würde und je nachdem wie angreifbar das Programm gegenüber Hackern ist.“

Ein weiteres Thema war, dass aus einem Datenmissbrauch negative Konsequenzen für die eigene Person, insbesondere im Gesundheitsbereich, entstehen könnten, z. B.:

„Also das ist, glaube ich, das Entscheidende, dass das nicht verkauft wird an irgendwelche Pharmaunternehmen oder dass man, wenn ich dann eine Lebensversicherung abschließen möchte, ich gesagt bekomme, du hast in der App aber eingetragen, dass du vielleicht irgendwie depressiv bist oder so was.“

Bedenken in diesem Bereich wurden teilweise geäußert im Zusammenhang mit der elektronischen Patientenakte und der damit verbundenen Befürchtung, dass Daten an Krankenkassen weitergegeben werden könnten. Generell wurden Gesundheitsdaten von einigen Teilnehmenden als sehr sensible, schützenswerte Daten beschrieben. Daraus resultiere dann eine geringe Intention, an einer Studie teilzunehmen, in der solche Daten geteilt werden müssten.

### Fähigkeiten, Überzeugungen und kontextuelle Bedingungen

In Hinblick auf Selbstwirksamkeitserwartungen (Domäne *Überzeugungen über Fähigkeiten*) gab es Teilnehmende, die generell optimistisch waren, eine Teilnahme realisieren zu können und die zeitlichen Ressourcen dafür aufbringen zu können. Es gab jedoch auch Teilnehmende, die sich nicht sicher waren, ob sie ihre Teilnahme zuverlässig gewährleisten könnten, insbesondere angesichts von Hindernissen wie zeitlichen und alltäglichen Herausforderungen und externen Ereignissen (Domäne *Umweltbedingungen und Ressourcen*). Krisensituationen wurden teilweise als ein Faktor angesehen, der die Teilnahme erschweren könnte. Andere Teilnehmende betonten, dass insbesondere Krisensituationen als einschneidende (saliente) externe Ereignisse sie zur Teilnahme motivieren würden.

Den Teilnehmenden war es zudem wichtig, die Kontrolle über ihre Studienteilnahme zu behalten. Dazu gehöre die Freiheit, zu bestimmen, wann Befragungen durchgeführt würden und wie viel in die Befragung investiert würde, außerdem keine Bevormundung durch die App/Studie zu erfahren und Mitgestaltungsmöglichkeiten durch beispielsweise Feedbackfunktionen zu erhalten. Zu diesem Kontrollbedürfnis gehörte auch, dass es einigen Teilnehmenden wichtig war, jederzeit darüber informiert zu sein, was sie bei einer Teilnahme erwartet. Das betrifft sowohl die konkreten Aufgaben für sie als Teilnehmende und die Dauer der Befragungen als auch den Studienzweck und die Verwendung der Ergebnisse.

### App-Gestaltung

Aussagen zu *kontextuellen Bedingungen und Ressourcen* der Teilnahme betrafen die App-Gestaltung und das Studiendesign.

Besonders wichtig schien die Nutzung vorhandener Technik: Die Teilnahme sollte auch bei eingeschränkter Internetkonnektivität, mit älteren Smartphones oder alternativ über den Computer möglich sein. Zudem wurde die Notwendigkeit einer mehrsprachigen und barrierearmen Gestaltung betont (z. B. angepasst an technische Kompetenzen, gute Lesbarkeit). Diese Anforderungen wurden wiederholt nicht nur für die eigene Nutzung, sondern auch im Sinne einer repräsentativen Beteiligung verschiedener Bevölkerungsgruppen diskutiert.

„Also ich glaube auch das Stichwort Mittelschicht, also Status, ist relevant. … viele Menschen, die ein Smartphone haben. Davon gibt es aber viele, die keine Internetanschlüsse haben, sondern nur Internet haben, wenn sie irgendwo ein öffentliches finden.“

Die Teilnehmenden betonten die Bedeutung einer transparenten Gestaltung der App. Eine klare und niedrigschwellige Dokumentation von Zielen und Abläufen innerhalb der Erhebungssoftware würde nicht nur eine gute Bedienbarkeit gewährleisten, sondern auch Vertrauen schaffen.

„… innerhalb der App auch irgendwie. Meistens gibt’s dann so ein Fragezeichen-Symbol, wo man sich dann mehr Infos reinholen kann. Also nicht nur wie die Daten verarbeitet werden. Ich glaube es ist so im Datenschutzgesetz aber, sondern halt auch wie wird sowas weiterverarbeitet? Inwieweit kann es helfen?“

Teilnehmende betrachteten digitale Erhebungsinstrumente als wertvolle Ressourcen, die durch gezielte Anpassungen, etwa bei Sprachbarrieren, eine breite Teilnahme ermöglichen könnten.

## Diskussion

### Zusammenfassende Darstellung der Ergebnisse

Das Konzept einer adaptiven und kontextsensitiven Echtzeitkohortenstudie in gesundheitlichen Krisenzeiten ist von den Teilnehmenden mit Interesse aufgenommen und diskutiert worden. Nach einer Beschreibung der Studie konnten sie klar formulieren, welche Erwartungen sie mit einer Teilnahme verknüpfen und welche Bedingungen ihnen wichtig wären.

Auf gesellschaftlicher Ebene wurden insbesondere das Mitwirken am Gemeinwohl, die Möglichkeit, eine Stimme abzugeben, und die Unterstützung politischer Entscheidungsprozesse durch Evidenz aus der PULS-Studie als Motive genannt. Persönliche Erwartungen umfassten Wissenszuwachs zu Gesundheitsthemen und verschiedene Formen der Aufwandsentschädigungen. Bei der Entscheidung für oder gegen eine Teilnahme standen nicht nur potenzielle Vorteile, sondern auch antizipierte Risiken im Fokus – insbesondere Datenschutzbedenken.

Die Teilnehmenden äußerten sich weiterhin zu ihren eigenen Kapazitäten im Hinblick auf eine Teilnahme. So sahen Teilnehmende Alltagspflichten, fehlende Technikexpertise und Sprachbarrieren als größte Hindernisse, konnten aber auch gut einschätzen, was sie für eine (fortlaufende) Teilnahme benötigten. Viele waren optimistisch, dass durch erleichternde Faktoren, wie eine einfache Sprache und flexible Ausfüllmöglichkeiten, eine Teilnahme ermöglicht werden könne.

Ein Großteil der Äußerungen der Befragten bezog sich auch auf ihre Wünsche und Anforderungen an die Studie. Dabei kam das Thema Fairness und Inklusion im Hinblick auf marginalisierte Gruppen wiederholt vor. Für Teilnehmende aus Minderheiten war dies teils eine entscheidende Teilnahmevoraussetzung. Andere Befragte betonten die Notwendigkeit einer repräsentativen Stichprobe und gaben dabei zu bedenken, die unterschiedlichen Fähigkeiten und Bedarfe verschiedener Personengruppen angemessen zu berücksichtigen. Der Aspekt, als *Public Health Sentinel* auch für andere Personengruppen, zum Beispiel Minderheiten, zu sprechen, wurde (kontrovers) diskutiert.

### Persönlicher und gesellschaftlicher Nutzen sowie Datenschutz als wichtige Voraussetzungen

Der persönliche und gesellschaftliche Nutzen einer Studienteilnahme war ein großes Thema in allen Fokusgruppen. Für einige wenige war vor allem der persönliche Nutzen ausschlaggebend – sei es in Form von monetärer Vergütung oder gesundheitlichen Vorteilen. Hier wurde deutlich, dass in Anbetracht vieler professioneller und kostenlos verfügbarer Smartphone-Apps der Anspruch an eine Studien-App hoch ist.

Im Zusammenhang mit dem gesellschaftlichen Nutzen wurde deutlich, dass die Einflussnahme der Studie auf Politik und Gesellschaft von hoher Relevanz für viele Teilnehmenden war. Dieses Thema wurde auch deswegen intensiv diskutiert, da eine große Skepsis darüber herrschte, inwieweit die PULS-Studie dies überhaupt leisten könne. In diesem Zusammenhang wurde deutlich, dass die Teilnehmenden ein gutes Verständnis davon hatten, wie ein optimal eingebetteter Forschungsprozess aussehen sollte: von einer aktiven Beteiligung der Bevölkerung vorab, einer repräsentativen Stichprobe und einem passenden Studiendesign, als Voraussetzung für eine hohe Aussagekraft der Daten, bis hin zur Translation und Implementierung in die realen Lebenswelten. Die Erwartungen der Teilnehmenden gingen über das hinaus, was in den meisten Fällen innerhalb einer Studie geleistet werden kann, und enthielten viele Ansprüche an eine evidenzbasierte Politikgestaltung, die bei den Bürger:innen anfängt. Die Teilnehmenden wünschten sich, ihrer Stimme Gehör zu verschaffen, und sahen in der PULS-Studie eine Möglichkeit (und teilweise eine Pflicht) dazu, sich gesellschaftlich zu beteiligen.

Das Thema Datenschutz wurde entsprechend der bestehenden Forschungsliteratur [3, 7, 11] auch in dieser Studie immer wieder als Barriere zur Teilnahme genannt. Über die bestehende Literatur hinaus konnte jedoch ein neuer interessanter Aspekt aufgedeckt werden: Bedenken gab es vor allem hinsichtlich der Kompetenz des Studienteams, die Daten vor Hackerangriffen und Missbrauch zu schützen. Gleichzeitig wurden die Reichweite des Einflusses und damit die sinnvolle Weiterverwendung der Teilnehmendendaten durch die Forschenden infrage gestellt.

### Einstellung zur eigenen Rolle als Public Health Sentinel

Über die Literatur zur Teilnahme an App-basierten Gesundheitsstudien hinaus ging es in unseren Fokusgruppen darum, die Einstellung dazu, sich als *Public Health Sentinel* zu identifizieren und in dieser Rolle gesundheitsrelevante Informationen zu teilen, zu untersuchen. Generell befürwortete ein Großteil der Befragten den Grundgedanken, durch die Teilnahme etwas für andere zu tun und zum Gemeinwohl beizutragen. Dies war auch eines der meistgenannten Ziele, das durch eine mögliche Teilnahme erreicht werden sollte. Eine regelmäßige Darstellung des eigenen Beitrags wurde als wichtige Verstärkung genannt, um eine Teilnahme über den Zeitraum von zwei Jahren aufrechtzuerhalten. Doch auch wenn sich die Befragten weitestgehend einig in der Bereitwilligkeit waren, ihre eigene Stimme für das Gemeinwohl zu nutzen, unterschied sich ihre Zustimmung bei dem Gedanken, auch über andere Personen in ihrem Umfeld Aussagen zu treffen. So stand ein Teil der Befragten der Idee positiv gegenüber. Insbesondere die Möglichkeit, Personen eine Stimme zu geben, die sich selbst nicht äußern können, fand dabei Anklang. Im Gegensatz dazu zweifelten manche an ihrer Fähigkeit, akkurate Aussagen über andere treffen zu können. Außerdem empfanden einige es als Ausspionieren, Aussagen über andere zu treffen, und lehnten dies entsprechend ab.

Zusammenfassend scheint die Entscheidung, ein *Public Health Sentinel* zu sein, nicht durch ein einfaches Ja oder Nein gefasst werden zu können. Allerdings ist eine Grundbereitschaft erkennbar, sich auf diese Weise für das Gemeinwohl aktiv einzusetzen.

### Komplexe Abwägungsprozesse

Ein zentraler Mehrwert unserer Fokusgruppen bestand darin, dass sie Einblick in die komplexen Abwägungsprozesse der Befragten im Hinblick auf eine Studienteilnahme ermöglichten. Viele der bereits genannten Aspekte ließen sich nicht eindeutig als Barrieren oder Anreize klassifizieren, sondern entfalteten ihre Bedeutung erst im Zusammenspiel mit anderen Faktoren – sie gewannen oder verloren je nach Kontext an Relevanz. Oft verliefen die Abwägungsprozesse an den Schnittstellen der Domänen: *Ziele, Erwartungen über Auswirkungen* und *Verstärkung*. Überschneidungen innerhalb dieser Domänen und mit anderen Bereichen wurden ebenfalls deutlich.

Häufig wurde abgewogen, dass der gesellschaftliche Nutzen der Studie Vorrang hätte, während persönliche, gesundheitliche oder finanzielle Vorteile eine geringere Rolle spielten. Auch waren die Teilnehmenden eher bereit, eine Vereinbarkeit mit ihren zeitlichen Verfügbarkeiten und Alltagsaufgaben zu ermöglichen, wenn die sichtbaren Auswirkungen der Studie sich mit den persönlichen Zielen für die Studie deckten. Wenn die Studie einen bedeutungsvollen Einfluss auf Politik oder Gesundheit hätte, würde ein höherer Studienaufwand z. B. durch Befragungsmenge und -zeiten geduldet. Auch Bedenken, dass eine Studienteilnahme für bestimmte Personengruppen aufgrund fehlender Kompetenzen oder alltagspraktischer Einschränkungen nicht möglich sei, könne durch Anpassungen des Studiendesigns oder technische Lösungen entgegengewirkt werden (z. B. Überwinden von Sprachbarrieren durch Spracheinstellungen in der App).

### Stärken und Limitationen

Zur Gewährleistung eines heterogenen Teilnehmendenpools und zur Reduzierung einer Überrepräsentation sehr forschungsinteressierter Teilnehmender haben wir uns für eine zeitintensive Rekrutierung über persönliche Ansprache entschieden. So konnte eine heterogene Gruppe an Personen unterschiedlichen Alters, Berufs- und Bildungsstatus akquiriert werden. Schüler:innen, Studierende, Personen mitten im Berufsleben und Rentner:innen waren vertreten. Durch einen *Social Sensing Approach* [15, 16], indem die Teilnehmenden auch als Sprachrohr für ihr soziales Umfeld befragt wurden, erhielten wir zusätzlich Informationen über sonst selten erreichte Gruppen (z. B. queerer Personenkreis, Personen mit körperlichen und psychischen Einschränkungen). Insbesondere die *Community-Leader-*Gruppe ermöglichte uns Einblicke in weitere Personenkreise.

Die Fokusgruppen fanden in deutscher Sprache statt. Personen ohne deutsche Sprachkenntnisse konnten daher nicht eingeschlossen werden, auch wenn diese einen großen Anteil der Bevölkerung ausmachen. Dank unserer Frage nach dem persönlichen Umfeld (*Social Sensing*) wurde dennoch mehrfach auf die Bedürfnisse von Personen mit Sprachbarrieren hingewiesen. Spezielle Bedarfe dieser Personengruppe müssen jedoch für nachfolgende Studien und die Ausgestaltung der PULS-App berücksichtigt werden.

## Fazit

Die Fokusgruppen legen nahe, dass unter gewissen Umständen die Teilnahme an einer umfangreichen und komplexen Studie wie der PULS-Studie akzeptabel und umsetzbar ist. Zentrale Aspekte sind hier vor allem verfügbare zeitliche Ressourcen, Datenschutz und Sicherheit, aber auch ein sichtbarer Einfluss auf Gesellschaft und Politik und eine Translation von Forschung in Politik. Diese in den Fokusgruppen identifizierten Determinanten einer Studienteilnahme werden nicht nur für die weitere Ausgestaltung der PULS-Studie wichtig, sondern können auch Planungsprozesse anderer Studien informieren. Dabei wird es insbesondere im Fall komplexerer (z. B. längsschnittlicher oder agiler) Studien wichtig sein, mögliche Schlüsselanreize für Bürger:innen zu nutzen.

## Supplementary Information

Als Supplementary Information sind eine Tabelle zur Zusammensetzung der Fokusgruppen (S1) und eine Tabelle zum Kodiermaterial (S2) beigefügt.

## Data Availability

Die während der vorliegenden Studie erzeugten und/oder analysierten Datensätze sind auf begründete Anfrage bei der Korrespondenzperson erhältlich.

## Footnotes

1 In einer folgenden Vignettenstudie wird eine passende Übersetzung des Public Health Sentinel ins Deutsche untersucht. Mit den Fokusgruppenteilnehmenden wurden verschiedene Begriffe diskutiert.

2 Die Videos finden sich über den Menüpunkt „Files“ in diesem Verzeichnis: https://osf.io/e9dz7/.
