# Supplementary material for: Citizens as public health sentinels: understanding study participation in an adaptive and context-sensitive real-time cohort study during times of crisis
Source: Bundesgesundheitsblatt Gesundheitsforschung Gesundheitsschutz. 2025 Jul 28;68(9):1035–44. [Article in German] doi: 10.1007/s00103-025-04108-3 (PMC12391147; doi:10.1007/s00103-025-04108-3)
Supplement: Supplementary file 1 — Als Supplementary Information sind eine Tabelle zur Zusammensetzung der Fokusgruppen (S1) und eine Tabelle zum Kodiermaterial (S2) beigefügt. [file 103_2025_4108_MOESM1_ESM.pdf]

Onlinematerial

Interviewleitfaden

Interviewleitfaden Fokusgruppen
Stand: 29.01.25

Setting:
2 Moderator/Moderatorinnen, (ggf. 1 Beobachter/Beobachterin der/die mitnotiert
Nach Hälfte der Zeit kann Beobachter/Beobachterin Zettel geben mit Punkten, die vom Moderator/Moderatorin ggf. vergessen wurden)

Bei Ankunft/ Warten:
Schön, dass Sie da sind. Solange wir auf die anderen warten, können Sie schon einmal die Einwilligungserklärung ausfüllen.

Herzlich Willkommen und schön, dass Sie heute hierher gefunden haben. Herzlichen Dank, dass Sie sich bereit erklärt haben, an unserer Fokusgruppendiskussion teilzunehmen.

Ich bin XX und das ist meine Kolleginnen XX.

In den nächsten 90 Minuten werden wir gemeinsam ein Gespräch zur Teilnahme an app-basierten Gesundheitsstudien durchführen. Dazu präsentieren wir Ihnen zuerst, was wir mit solchen app-basierten Gesundheitsstudien meinen und laden Sie dann einschließend ein, Ihre Meinung mit uns zu teilen. Die Idee ist, dass wir ein offenes Gespräch miteinander führen und uns jede Meinung interessiert.

Alles, was Sie uns erzählen, ist ein wichtiger Beitrag für unsere Forschung. Es gibt keine falschen Antworten. Deshalb möchten wir Sie bitten, offen und frei zu sprechen. Wir möchten Sie auch ermutigen, Ihre Meinung zu teilen, wenn sie von anderen abweicht. Wir möchten Sie auch ermutigen miteinander zu sprechen und gerne auf das Gesagte voneinander einzugehen.

Ich werde zwischendrin Leitfragen stellen und das Gespräch moderieren. Für unsere Diskussion ist es noch wichtig, dass Sie einander ausreden lassen und einander respektvoll begegnen.

Noch ein paar Punkte gibt es zu beachten, die ausführlich auch nochmal in der Studieninformation stehen:

- Ihre Teilnahme ist freiwillig.
- Sie können jederzeit die Studie abbrechen und das Gespräch verlassen.
- Für Auswertungszwecke werden wir das Gespräch aufzeichnen. Sobald die Gespräche transkribiert sind, werden diese Aufzeichnungen direkt gelöscht. Ihre Namen werden nirgends gespeichert.
- Wenn es für Sie in Ordnung ist, werden wir auch ein Video mitlaufen lassen. Das erleichtert uns nachher, das Gesagte zu transkribieren. Auch dieses Video wird direkt nach der Transkription gelöscht.
- Ihre Daten sind bei uns geschützt, werden streng vertraulich behandelt und nur in anonymisierter Form weiter verwendet.

Wenn Sie mit der Teilnahme einverstanden sind, bitten wir Sie, noch die Einwilligungserklärung noch zu unterzeichnen, falls Sie das noch nicht getan haben.

Wenn es für Sie in Ordnung ist, würden wir uns alle für die Dauer dieser Gesprächsrunde mit Vornamen ansprechen.

Kategorie	Frage(n)		Follow-Up Frage	Erklärung
Ice-Breaker und Transition; Teilnehmende ins Sprechen bringen	***Am Anfang würden wir mit einer kurzen Vorstellungsrunde beginnen. Sagen Sie bitte in einem Satz Ihren Vornamen, woher Sie kommen und Ihr Lieblingsessen.***			
	Bevor wir ins Thema starten, wollen wir kurz einen Rückblick zur COVID-19-Pandemie machen.<br><br>***Wie haben Sie damals die Maßnahmen in Ihrem Alltag wahrgenommen?***<br>***Was hätten Sie sich damals persönlich gewünscht?***			Ziel: Emotionale Aufladung
			Was lief für Sie persönlich gut oder schlecht?	
	Jetzt haben wir schon einige Ideen von Ihnen bekommen.			
	**VIDEO 1 Narrativ/Ziel**			
	Was denken Sie spontan über die Teilnahme an einer solchen Studie?<br><br>***...denken Sie da gerne kurz drüber nach und merken sich Ihre Gedanken. Bevor wir darüber sprechen, zeigen wir Ihnen noch ein kurz ein Video, in dem die Studie konkreter beschrieben wird.***			Vielleicht erst mal nur einen schnellen Satz/Gedanken zulassen<br><br>Und dann die Wächter/Hüter-Frage anschließen
	**Video 2: Eigenschaften**			
	Was denken Sie spontan über die Teilnahme an einer solchen Studie?			
			Würden Sie mitmachen wollen? Warum/Warum nicht?	

	Wie finden Sie die Vorstellung ein Wächter, eine Wächterin oder Hüter/Hüterin der Gesundheit im Land zu sein? Was verbinden Sie damit?	
Themenfeld: Eigene Teilnahme	***Wie würde eine solche Studie in Ihren Alltag passen?***	Erläuterung: Gibt es irgendwas an der Studie, dass als total unpassend in einen normalen Arbeitsalltag wahrgenommen wird? Ist es realistisch, das so lange zu machen? Ist die Vorstellung, dass über das eigene Smartphone zu machen, in Ordnung oÄ<br><br>• Wichtig: Teilnehmer:innen sollen sich nicht zu sehr im Detail über die Studie verlieren. Die Rahmenbedingungen, die im Video gezeigt werden, sind unveränderlich.<br>• Auch wichtig: Wenn Teilnehmer:innen einen Aspekt der Studie kategorisch ablehnen, nochmal nachhaken, ob dieser Punkt wirklich ein hartes Kriterium ist, nicht teilzunehmen oder ob es

			z.B. irgendwelche anderen Rahmenbedingungen besser machen würden.
	Könnten Sie sich in Ihrem Alltag auch vorstellen, Empfehlungen nachzukommen, die Sie von der App bekommen?<br>***Also beispielsweise, dass Sie über die App eine Empfehlung bekommen, bei einer Hitzewelle zu Hause zu bleiben?***		
		Würden Sie das vielleicht sogar begrüßen?	
	Was könnte Sie zu einer Teilnahme bewegen?		Erläuterung: Hier ist die Idee, eher die positiven Aspekte herauszubekommen.
		• *Was fänden Sie denn* ***gut*** *daran?*<br>• *Was fänden/finden Sie* ***sinnvoll****?*<br>• *Warum hätten Sie das Gefühl, dass eine Teilnahme an einer solchen Studie* ***sinnhaft*** *wäre?*<br>• *Welche* ***Aufwandsentschädigung*** *fänden Sie angemessen? Welche Form: Geld, Spenden, Gutscheine, Gewinnspiel*<br>• *Was würde Sie motivieren, über einen* ***langen Zeitraum*** *am Ball zu bleiben?*	

<table>
<tr><td rowspan="2">Themenfeld: Meinung des Umfelds</td><td colspan="2">Sie sind hier (vor Ort) und nehmen an unserer Gruppendiskussion teil und sind damit auch motiviert einen Beitrag zur Forschung zu leisten/zeigen damit Motivation und Interesse.
Wie sieht das denn in Ihrem Freundes- und Bekanntenkreis oder Familie aus, wie wäre dort die Motivation, an einer solchen Studie/Studien teilzunehmen?

Wer würde teilnehmen, und wer eher nicht?

Und vor allem: warum?</td><td>Wer in Ihrem sozialen Umfeld würde mitmachen?</td></tr>
<tr><td></td><td>Und warum?
Welchen Grund hätten die einzelnen Personen auf Familien-/ Freundes-/ Bekanntenkreis, teilzunehmen oder nicht teilzunehmen?
Was wären wichtige Aspekte für die Familie/Freunde/Bekannte?
Wo haben Sie das Gefühl stimmen Sie mit Ihrem Umfeld überein und was sehen Sie vielleicht anders / würde Sie eher zur Teilnahme bewegen als ihr Umfeld?
Wie würden Ihre Freunde es beurteilen, wenn Sie teilnehmen?</td><td>Erläuterung: Allgemeine „Verträglichkeit“ und Akzeptanz der Umfrage über die Fokusgruppenteilnehmer:innen hinaus, hier evtl. die Möglichkeit Sachen zu sagen, die man nicht so gerne über sich selbst sagen würde (z.B. Skepsis äußern?)

Hier versuchen, die unterschiedlichen Gruppen aus dem Umfeld aufzugreifen also die Gründe derer, die teilnehmen und derer, die nicht teilnehmen. Evtl. unterschiedliche Personengruppen (Leute mit Kindern, Freunde, Eltern…)</td></tr>
</table>

		• ***Wenn Sie am Tisch mit Freunden/Familie sitzen, wie würden Sie über diese geplante Studie vielleicht erzählen. Was würde zurückkommen?***	
**ZUSAMMENFASSUNG DER PUNKTE: Vielen Dank für Ihre ganzen tollen Ideen und Gedanken. Ich fasse nochmal kurz zusammen und Sie melden mir zurück, ob ich noch etwas wichtiges vergessen habe. Moderator: Bitte ergänz auch noch, wenn dir noch etwas einfällt.**			
Ending Question	***Wenn Sie jetzt nochmal an den Anfang und an die Erfahrungen in der Corona-Zeit denken.***<br>***Was würden Sie sich durch eine solche Studie/App erhoffen?***<br>***Was denken Sie, könnte und sollte durch eine solche Studie anders laufen?***<br><br>***Was wäre Ihnen als Teilnehmer/Teilnehmerin besonders wichtig?***<br>***Wurde etwas genannt, was Sie besonders unterstreichen würden?***<br>***Oder wurde etwas nicht genannt was Ihnen wichtig wäre?***		
		• ***Wenn noch Zeit:*** *Wenn Sie noch kurz Zeit haben/ Wer noch kurz Zeit hat: Möchten Sie uns noch kurz Feedback zu dieser Gesprächsgruppe geben? Was fanden Sie gut und was könnten wir noch besser machen?*	Erläuterung: Hier nochmal versuchen zu greifen, was den Teilnehmer:innen am wichtigsten ist oder war. Hier sollen auch noch Sachen Platz finden können, die in den Fragen oben evtl. keinen Raum hatten.

Wichtige allgemeine Punkte:

- nicht zu sehr „abdriften“ in Modalitäten der Studie – Einfangen, in dem man sagt, dass das Video bisher eine Skizze der Studie ist, genaueres ist noch nicht klar
- die Modalitäten der Studie sind unveränderlich (z.B. Zeitraum, Befragungsfrequenz). Hier nicht zu sehr drin verlieren.
- Darauf achten, alle Personen einzubeziehen – manchmal auf die Leute eingehen, die wenig sagen
- **Neutrale** Haltung, eigene Meinung nicht reinbringen
- Moderierende Person hat vor allem eine Rolle als **aktiver Zuhörer**
- Rückfrage, wenn jemand etwas sagt „Wie sehen das die anderen?“
- Zusammenfassung zwischendurch von dem was gesagt wurde oder rückfragen „Habe ich das richtig verstanden?“

Gesprächsleitlinie:
Paraphrasieren, Validieren, aktives Zuhören

Sätze für Nachfragen:

- Fällt Ihnen noch etwas ein?

Tabelle S1: Zusammensetzung und Eigenschaften Fokusgruppen

Fokusgruppe	Gruppengröße	Dauer hh:mm	Altersrange (Jahre)	Darbietung	Leitung / Anwesend	Berufe/Berufsbezeichnungen der Teilnehmer:innen	Codierung
1	7	01:23	22 - 42*	Präsenz	M1: KH,<br>M2: AKK<br>B: SZ	Polizist:in, Student:innen (2), Consultant, NA, Versicherungskaufmann/frau, Casemanager:in	SZ, Hiwi
2	6	01:21	21 - 71	Präsenz	M1: KH,<br>M2: BM	Rentner:innen (2), Azubi Kaufmann/Kauffrau Groß- und Einzelhandel, Außendienstler:in, Kommunikationsmanager:in	HZH, Hiwi
3	8	01:35	29 - 72	Präsenz	M1: BK<br>M2: AKK<br>B: HZH	HR/Vertrieb, Kommissionierer:in, Staatsanwält:in, Informatiker:in, Hausfrau, Direktor:in, Friseur:in/Student:in	SZ, Hiwi
4	5	01:13	19 - 59	Online	M1: SZ<br>M2: HZH	Sekretär:in, Schüler:innen (2),	HZH, Hiwi
5	6	01:28	19 - 82	Präsenz	M1: KH<br>M2: AKK	Schüler:in, Arbeitssuchend, Ehrenamt, Rentner:in, Verkäufer:in	CJ, SZ
6	4	01:24	19 - 65*	Präsenz	M1: BK<br>M2: AKK	FSJ-ler, MRTA, Schauspieler:in	HZH, Hiwi
7	6	01:27	26 - 60*	Online	M1: BK<br>M2: BM<br>(SZ zur Begrüßung)	Elektroingenieuer:in, Angestellte:r im öffentlichen Dienst (3), Schulleiter:in, Bürgermeister:in/Jurist:in,	SZ, HZH

**von einer Person aus der Gruppe fehlt die Altersangabe.*

Tabelle S2: Kodiermanual entsprechend der Kategorien des Theoretical Domains Framework

**Domain**	Construct
Wissen	Wissen<br>*Ein Bewusstsein für die Existenz von etwas.*
	Prozedurales Wissen<br>*Wissen, wie man etwas tut. (implizit)*
	Wissen über die Aufgabenumgebung<br>*Wissen über den sozialen und materiellen Kontext, in dem eine Aufgabe durchgeführt wird.*
Fähigkeiten und Kompetenzen	Fähigkeit<br>*Eine durch Training und/oder Übung erworbene Fähigkeit oder Fertigkeit.*
	Fähigkeitsentwicklung<br>*Der allmähliche Erwerb oder Fortschritt durch fortschreitende Stadien einer durch Training und Übung erworbenen Fähigkeit oder Fertigkeit.*
	Kompetenz<br>*Das Repertoire an Fähigkeiten und Fertigkeiten einer Person, insbesondere wie sie auf eine bestimmte Aufgabe oder Aufgaben angewendet werden.*
	Fähigkeit<br>*Kompetenz oder Kapazität, eine physische oder mentale Handlung auszuführen. Fähigkeit kann entweder angeboren oder durch Bildung und Praxis erworben sein.*
	Zwischenmenschliche Fähigkeiten<br>*Eine Begabung, die es einer Person ermöglicht, effektive Beziehungen zu anderen zu führen, wie z. B. die Fähigkeit zur Zusammenarbeit, zur Übernahme angemessener sozialer Verantwortlichkeiten oder*

	zur Ausstellung angemessener Flexibilität.
	Übung<br>*Wiederholung einer Handlung, eines Verhaltens oder einer Reihe von Aktivitäten, oft zur Verbesserung der Leistung oder zum Erwerb einer Fähigkeit.*
	Fähigkeitsbewertung<br>*Eine Beurteilung der Qualität, des Wertes oder des Niveaus einer durch Training und Praxis erworbenen Fähigkeit.*
Soziale/berufliche Rolle und Identität	Professionelle Identität<br>*Die Merkmale, durch die eine Person in Bezug auf einen bestimmten Beruf erkannt wird.*
	Professionelle Rolle<br>*Das Verhalten, das für eine bestimmte Art von Arbeit oder soziale Position als angemessen angesehen wird.*
	Identität<br>*Das Selbstverständnis einer Person, definiert durch a) eine Reihe physischer und psychologischer Merkmale, die nicht vollständig mit einer anderen Person geteilt werden, und b) eine Reihe sozialer und zwischenmenschlicher Zugehörigkeiten (z. B. Ethnizität) und sozialer Rollen.*
	Professionelle Grenzen<br>*Die Grenzen oder Limits, die sich auf einen bestimmten Beruf oder eine Berufung beziehen.*
	Professionelles Selbstvertrauen<br>*Der Glaube einer Person an ihr Repertoire an Fähigkeiten und Fertigkeiten, insbesondere wie sie auf eine bestimmte Aufgabe oder Aufgaben angewendet werden.*
	Gruppenidentität<br>*Die Verhaltensweisen oder persönlichen Merkmale, durch die eine Person als Mitglied einer Gruppe*

	erkennbar ist.
	Führung<br>*Die Prozesse, die das Führen anderer beinhalten, einschließlich Organisation, Leitung, Koordination und Motivation ihrer Bemühungen zur Erreichung bestimmter Gruppen- oder Organisationsziele.*
	Organisationale Bindung<br>*Die Hingabe eines Mitarbeiters an eine Organisation und der Wunsch, Teil davon zu bleiben. Organisatorisches Engagement wird oft so beschrieben, dass es sowohl eine emotionale oder moralische als auch eine eher pragmatische Komponente hat.*
Überzeugungen über Fähigkeiten	Selbstvertrauen<br>*Selbstvertrauen/Selbstsicherheit oder Vertrauen in die eigenen Fähigkeiten, Fertigkeiten und das eigene Urteilsvermögen.*
	Wahrgenommenes Vertrauen<br>*Der Glaube einer Person an ihre Fähigkeit, Fähigkeiten zu erlernen und auszuführen.*
	Selbstwirksamkeit<br>*Die Fähigkeit einer Person, effektiv zu handeln, um die gewünschten Ergebnisse zu erzielen (wie es vom Einzelnen wahrgenommen wird)*
	Wahrgenommene Verhaltenskontrolle<br>*Die Wahrnehmung einer Person über die Leichtigkeit oder Schwierigkeit, ein bestimmtes Verhalten auszuführen.*
	Überzeugungen<br>*Die als wahr gehaltenen Aussagen oder Propositionen.*
	Selbstwertgefühl<br>*Das Maß, in dem die Qualitäten und Merkmale des Selbstkonzepts als positiv wahrgenommen werden.*

	Ermächtigung<br>*Die Förderung der Fähigkeiten, des Wissens und des Vertrauens, die notwendig sind, um mehr Kontrolle über das eigene Leben zu übernehmen, wie in bestimmten Bildungs- oder Sozialprogrammen; die Delegation erweiterter Entscheidungsbefugnisse an Einzelpersonen oder Gruppen in einer Gesellschaft oder Organisation.*
	Berufliches Selbstvertrauen<br>*Der Glaube eines Individuums an sein Repertoire an Fähigkeiten und Fertigkeiten, insbesondere wie es auf eine Aufgabe oder eine Reihe von Aufgaben angewendet wird.*
Optimismus	Optimismus<br>*Die Einstellung, dass Ergebnisse positiv sein werden und dass die Wünsche oder Ziele der Menschen letztendlich erfüllt werden.*
	Pessimismus<br>*Die Einstellung, dass Dinge schiefgehen werden und dass die Wünsche oder Ziele der Menschen wahrscheinlich nicht erfüllt werden.*
	Unrealistischer Optimismus<br>*Die angeborene Tendenz des Menschen, die eigenen Fähigkeiten und Chancen auf positive Ergebnisse im Vergleich zu denen anderer Menschen zu überschätzen.*
	Identität<br>*Das Selbstverständnis einer Person, definiert durch a) eine Reihe physischer und psychologischer Merkmale, die nicht vollständig mit einer anderen Person geteilt werden, und b) eine Reihe sozialer und zwischenmenschlicher Zugehörigkeiten (z. B. Ethnizität) und sozialer Rollen.*
Erwartungen über Auswirkungen	Überzeugungen<br>Das, was geglaubt wird; die Aussage oder der Satz von Aussagen, die als wahr gehalten werden.
	Ergebnis-Erwartungen

	Kognitive, emotionale, verhaltensbezogene und affektive Ergebnisse, von denen angenommen wird, dass sie mit zukünftigen oder beabsichtigten Verhaltensweisen verbunden sind. Diese angenommenen Ergebnisse können zukünftige Verhaltensweisen entweder fördern oder hemmen.
	Merkmale von Ergebnis-Erwartungen<br>*Merkmale der kognitiven, emotionalen und verhaltensbezogenen Ergebnisse, von denen Individuen glauben, dass sie mit zukünftigen oder beabsichtigten Verhaltensweisen verbunden sind und die entweder diese Verhaltensweisen fördern oder hemmen. Dazu gehören, ob sie Sanktionen/Belohnungen, proximal/distal, wertgeschätzt/nicht wertgeschätzt, wahrscheinlich/unwahrscheinlich, salient/nicht salient, wahrgenommene Risiken oder Bedrohungen sind.*
	Erwartete Reue<br>*Ein Gefühl der potenziellen negativen Konsequenzen einer Entscheidung, das die getroffene Wahl beeinflusst: Zum Beispiel kann ein Individuum entscheiden, keine Investition zu tätigen, aufgrund der Gefühle, die mit einem vorgestellten Verlust verbunden sind.*
	Konsequenzen<br>*Ein Ergebnis eines Verhaltens in einer bestimmten Situation.*
Verstärkung	Belohnungen (proximal/distal, wertgeschätzt/nicht wertgeschätzt, wahrscheinlich/unwahrscheinlich)<br>*Rückgabe oder Entschädigung, die einer Person für eine bestimmte Leistung gemacht oder von ihr erhalten wird.*
	Anreize<br>*Ein äußerer Reiz, wie eine Bedingung oder ein Objekt, das das Verhalten fördert oder als Motiv dient.*
	Bestrafung<br>*Der Prozess, bei dem die Beziehung zwischen einer Reaktion und einem Stimulus oder Umstand dazu führt, dass die Reaktion weniger wahrscheinlich wird; ein schmerzhaftes, unerwünschtes oder unerwünschtes Ereignis oder Umstand, das/die als Strafe für einen Fehlverhalten auferlegt wird.*

	Konsequenzen<br>*Ein Ergebnis eines Verhaltens in einer bestimmten Situation.*
	Verstärkung<br>*Ein Prozess, bei dem die Häufigkeit einer Reaktion durch eine abhängige Beziehung oder Kontingenz mit einem Stimulus erhöht wird.*
	Kontingenzen<br>*Eine bedingte probabilistische Beziehung zwischen zwei Ereignissen. Kontingenzen können durch Abhängigkeiten arrangiert werden oder zufällig entstehen.*
	Sanktionen<br>*Eine Strafe oder andere Zwangsmaßnahme, die in der Regel von einer anerkannten Autorität verhängt wird, um unangemessene oder unautorisierte Handlungen zu bestrafen und zu verhindern.*
Intentionen	Stabilität von Absichten<br>*Die Fähigkeit, trotz störender Einflüsse an einem Entschluss festzuhalten.*
	Stufenmodell der Verhaltensänderung<br>*Ein Modell, das vorschlägt, dass Verhaltensänderung durch fünf spezifische Stadien erreicht wird: Vorüberlegung, Überlegung, Vorbereitung, Handlung und Aufrechterhaltung.*
	Transtheoretisches Modell und Stadien der Veränderung<br>*Eine Theorie mit fünf Stadien zur Erklärung von Veränderungen im Gesundheitsverhalten von Menschen. Sie legt nahe, dass Veränderung Zeit braucht, dass verschiedene Interventionen in verschiedenen Stadien wirksam sind und dass es mehrere Ergebnisse gibt, die über die Stadien hinweg auftreten.*
Ziele	Ziele (distal/proximal)<br>*Ein gewünschter Zustand einer Person oder eines Systems, der näher (proximal) oder weiter entfernt (distal) sein kann.*

	Zielpriorität<br>*Die Reihenfolge der Wichtigkeit oder Dringlichkeit von Endzuständen, auf die man hinarbeitet.*
	Ziel- / Zielsetzung<br>*Ein Prozess, der spezifische, zeitlich festgelegte Verhaltensziele festlegt, die messbar, erreichbar und realistisch sind.*
	Ziele (autonom / kontrolliert)<br>*Der Endzustand, auf den man hinarbeitet: der Zweck einer Aktivität oder Unternehmung. Er kann daran erkannt werden, dass eine Person ihr Verhalten einstellt oder ändert, wenn sie diesen Zustand erreicht hat; Beherrschung einer Aufgabe, die innerhalb eines festgelegten Zeitraums erreicht werden soll.*
	Aktionsplanung<br>*Die Handlung oder der Prozess, einen Plan in Bezug auf etwas zu erstellen, das zu tun ist oder eine Tat.*
	Implementierungsabsicht<br>*Der Plan, den man im Voraus darüber erstellt, wann, wo und wie man ein Verhalten ausführen wird.*
Gedächtnis, Aufmerksamkeits- und Entscheidungsprozesse	Gedächtnis<br>*Die Fähigkeit, Informationen oder eine Darstellung einer vergangenen Erfahrung zu behalten, basierend auf den mentalen Prozessen des Lernens oder Kodierens, der Beibehaltung über ein bestimmtes Zeitintervall und der Wiederherstellung oder Reaktivierung des Gedächtnisses; spezifische Informationen über eine bestimmte Vergangenheit.*
	Aufmerksamkeit<br>*Ein Zustand des Bewusstseins, in dem die Sinne selektiv auf Aspekte der Umgebung fokussiert sind und das zentrale Nervensystem in einem Zustand der Bereitschaft ist, auf Reize zu reagieren.*
	Aufmerksamkeitskontrolle<br>*Das Ausmaß, in dem eine Person sich auf relevante Hinweise konzentrieren und alle irrelevanten*

	Hinweise in einer gegebenen Situation ignorieren kann.
	Entscheidungsfindung<br>*Der kognitive Prozess der Auswahl zwischen zwei oder mehr Alternativen, die von relativ klar bis komplex reichen.*
	Kognitive Überlastung / Müdigkeit<br>*Die Situation, in der die Anforderungen, die durch mentale Arbeit an eine Person gestellt werden, größer sind als die mentalen Fähigkeiten der Person.*
Umweltbedingungen und Ressourcen	Umweltstressoren<br>*Externe Faktoren in der Umgebung, die Stress verursachen.*
	Ressourcen / materielle Ressourcen<br>*Rohstoffe und menschliche Ressourcen, die zur Ausführung eines Verhaltens verwendet werden.*
	Organisationskultur / -klima<br>*Ein charakteristisches Muster von Gedanken und Verhalten, das von Mitgliedern derselben Organisation geteilt wird und sich in ihrer Sprache, ihren Werten, Einstellungen, Überzeugungen und Gewohnheiten widerspiegelt.*
	Hervorstechende Ereignisse / kritische Zwischenfälle<br>*Vorkommnisse, die man als besonders, herausragend oder anderweitig bedeutsam beurteilt.*
	Person-Umwelt-Interaktion<br>*Wechselwirkung zwischen dem Individuum und seiner Umgebung.*
	Barrieren und Förderfaktoren<br>*In psychologischen Kontexten sind Barrieren/Förderfaktoren mentale, emotionale oder verhaltensbezogene Begrenzungen/Stärken von Individuen oder Gruppen.*
Soziale Einflüsse	Sozialer Druck

	Die Ausübung von Einfluss auf eine Person oder Gruppe durch eine andere Person oder Gruppe.
	Soziale Normen<br>*Sozial bestimmte, konsensuelle Standards, die anzeigen a) welche Verhaltensweisen in einem gegebenen Kontext als typisch gelten und b) welche Verhaltensweisen in diesem Kontext als angemessen angesehen werden.*
	Gruppenkonformität<br>*Die Handlung, bewusst einen bestimmten Grad an Ähnlichkeit zu denjenigen in den eigenen sozialen Kreisen aufrechtzuerhalten.*
	Soziale Vergleiche<br>*Der Prozess, durch den Menschen ihre Einstellungen, Fähigkeiten oder Leistungen im Vergleich zu anderen bewerten.*
	Gruppennormen<br>*Jedes Verhalten, jede Überzeugung, Einstellung oder emotionale Reaktion, die von einer bestimmten Gruppe in der Gesellschaft als korrekt oder akzeptabel angesehen wird.*
	Soziale Unterstützung<br><br>*Die Wahrnehmung oder Bereitstellung von Hilfe oder Trost für andere, typischerweise, um ihnen beim Umgang mit einer Vielzahl von biologischen, psychologischen und sozialen Belastungen zu helfen. Unterstützung kann aus jeder zwischenmenschlichen Beziehung im sozialen Netzwerk eines Individuums entstehen, einschließlich Freunden, Nachbarn, religiösen Institutionen, Kollegen, Pflegepersonen oder Unterstützungsgruppen.*
	Macht<br>*Die Fähigkeit, andere zu beeinflussen, selbst wenn sie versuchen, diesem Einfluss zu widerstehen.*
	Intergruppenkonflikt

	Unstimmigkeit oder Konfrontation zwischen zwei oder mehr Gruppen und deren Mitgliedern. Dies kann körperliche Gewalt, zwischenmenschliche Zwistigkeiten oder psychologische Spannungen beinhalten.
	Entfremdung<br>*Entfremdung von der eigenen sozialen Gruppe; ein tief verwurzeltes Gefühl der Unzufriedenheit mit den eigenen persönlichen Erfahrungen, das eine Quelle des Misstrauens gegenüber der sozialen oder physischen Umwelt oder sich selbst sein kann; die Erfahrung der Trennung zwischen Gedanken und Gefühlen.*
	Gruppenidentität<br>*Die Menge an Verhaltens- oder persönlichen Merkmalen, durch die eine Person als Mitglied einer Gruppe erkennbar ist [und sich als solche darstellt].*
	Modelllernen<br>*Im entwicklungspsychologischen Sinn der Prozess, bei dem ein oder mehrere Individuen oder andere Entitäten als Beispiele (Modelle) dienen, die ein Kind nachahmen wird.*
Emotionen	Angst<br>*Eine intensive Emotion, die durch die Wahrnehmung einer unmittelbar bevorstehenden Bedrohung ausgelöst wird und eine unmittelbare Alarmreaktion beinhaltet, die den Organismus durch eine Reihe physiologischer Veränderungen mobilisiert.*
	Besorgnis<br>*Ein Gefühlszustand, der durch Besorgnis und somatische Symptome von Anspannung gekennzeichnet ist, in dem ein Individuum eine bevorstehende Gefahr, Katastrophe oder ein Unglück erwartet.*
	Affekt<br>*Eine Erfahrung oder ein Gefühl von Emotion, das von Leiden bis zur Hochstimmung reicht, von den einfachsten bis zu den komplexesten Empfindungen von Gefühlen und von den normalsten bis zu den*

	pathologischsten emotionalen Reaktionen.
	Stress<br>*Ein Zustand physiologischer oder psychologischer Reaktion auf interne oder externe Stressoren.*
	Depression<br>*Ein mentaler Zustand, der mit gedrückter Stimmung, Interessen- oder Freudlosigkeit, Schuldgefühlen oder geringem Selbstwertgefühl, gestörtem Schlaf oder Appetit, geringer Energie und schlechter Konzentration einhergeht.*
	Positiver / negativer Affekt<br>*Der innere Gefühlszustand, der auftritt, wenn ein Ziel erreicht/nicht erreicht wurde, eine Bedrohung vermieden/nicht vermieden wurde oder die Person mit dem gegenwärtigen Zustand der Dinge zufrieden/nicht zufrieden ist.*
	Burn-out<br>*Physische, emotionale oder mentale Erschöpfung, insbesondere im Berufsleben, einhergehend mit verminderter Motivation, reduzierter Leistungsfähigkeit und negativen Einstellungen gegenüber sich selbst und anderen.*
Verhaltensregulation	Selbstbeobachtung<br>*Eine Methode im Verhaltensmanagement, bei der Individuen ein Protokoll ihres Verhaltens führen, insbesondere im Zusammenhang mit dem Versuch, sich selbst zu verändern oder zu regulieren; ein Persönlichkeitsmerkmal, das die Fähigkeit widerspiegelt, das eigene Verhalten in Reaktion auf Situationen zu modifizieren.*
	Gewohnheiten durchbrechen<br>*Ein Verhalten oder eine Abfolge von Verhaltensweisen zu beenden, das/die automatisch durch relevante situative Hinweise ausgelöst wird.*

	Aktionsplanung<br>*Die Handlung oder der Prozess, einen Plan in Bezug auf etwas zu erstellen, das zu tun ist oder eine Tat.*
